# Supplementary material for: Reducing sexual predation and victimization through warnings and awareness among high-risk users
Source: J Comput Soc Sci. 2025 Jun 29;8(3):70. doi: 10.1007/s42001-025-00399-3 (PMC12206673; doi:10.1007/s42001-025-00399-3)
Supplement: Supplementary file 1 — (pdf 184 KB) [file 42001_2025_399_MOESM1_ESM.pdf]

Supplementary Information

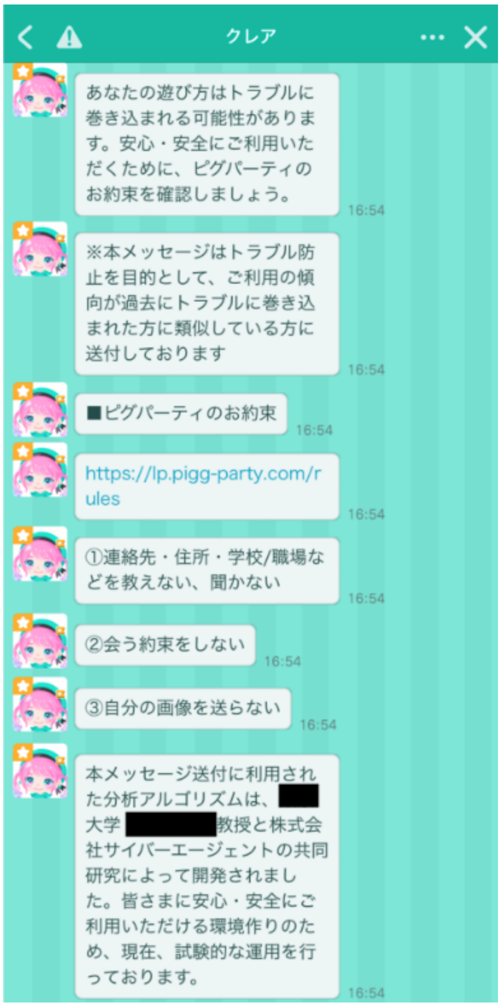

Fig. S1 Warning and awareness building message from the Pigg Party administrator (original Japanese version)
